# Impact of immune checkpoint inhibitors on survival outcomes in advanced gastric cancer in Japan: A real‐world analysis

**DOI:** 10.1002/cam4.7401

**Published:** 2024-06-20

**Authors:** Toru Kadono, Satoru Iwasa, Toshiharu Hirose, Hidekazu Hirano, Natsuko Okita, Hirokazu Shoji, Atsuo Takashima, Ken Kato

**Affiliations:** ^1^ Department of Gastrointestinal Medical Oncology National Cancer Center Hospital Tokyo Japan; ^2^ Cancer Chemotherapy Center Osaka Medical and Pharmaceutical University Takatsuki Osaka Japan

**Keywords:** gastric cancer, immune checkpoint inhibitors, overall survival, prognostic factors, real‐world data, time to treatment‐discontinuation

## Abstract

**Background:**

Nivolumab was approved for the treatment of advanced gastric cancer in 2017 in Japan. The aim of this study was to assess the impact of nivolumab in a real‐world clinical setting.

**Methods:**

This single‐institutional retrospective study included patients with advanced gastric or esophagogastric junction adenocarcinoma and a history of first‐line chemotherapy with platinum‐based doublet or triplet regimens between 2010 and 2020. To assess the impact of nivolumab on survival, the patients were divided based on the year of nivolumab approval into a pre‐2017 (2010–2016) group and a post‐2017 (2017–2020) group.

**Results:**

From a total of 1918 patients, 1093 were excluded. There were 533 patients in the pre‐2017 group and 292 in the post‐2017 group. Immune checkpoint inhibitors were used significantly more often in the post‐2017 group than in the pre‐2017 group (8.6% vs. 47.9%). Median overall survival was significantly longer in the post‐2017 group (16.9 vs. 13.9 months; hazard ratio [HR] 0.75, 95% confidence interval [CI] 0.63–0.90; *p* < 0.01). The proportion of patients transitioning to third‐line treatment was higher in the post‐2017 group than in the pre‐2017 group (56.3% vs. 43.8%, *p* < 0.01). Median survival outcomes following progression on second‐line treatment were significantly longer in the post‐2017 group (4.3 vs. 3.2 months; HR 0.70, 95% CI 0.57–0.86; *p* < 0.01).

**Conclusion:**

The proportion of patients transitioning to third‐line treatment and survival outcomes following progression on second‐line treatment have improved since the approval of nivolumab. This drug might help to prolong overall survival in real‐world practice.

## Graphical abstract

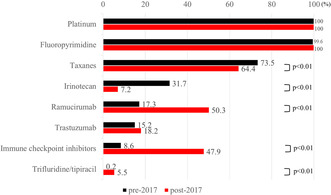

## 1 INTRODUCTION

Gastric cancer had the fifth highest incidence and fourth highest mortality rate of all the cancers worldwide in 2020.^1^ In Japan, it was ranked third for incidence in 2019 and for mortality in 2021.^2^ The prognosis for patients with gastric cancer with distant metastases remains dismal, with a 5‐year relative survival rate of 6.2% in Japan.^2^

In the 2000s, tegafur/gimeracil/oteracil potassium (S‐1), paclitaxel, docetaxel, and irinotecan were used for advanced gastric cancer (AGC) in Japan. Subsequently, many clinical trials were conducted, and several new drugs were approved in the 2010s. Trastuzumab, an anti‐human epidermal growth factor receptor‐2 (HER2) antibody, showed efficacy in HER2‐positive AGC in the ToGA trial,^3^ leading to its approval in Japan in 2011. In terms of second‐line treatment, the RAINBOW trial confirmed that ramucirumab, an anti‐vascular endothelial growth factor receptor‐2 antibody, was superior in combination with paclitaxel than paclitaxel alone; this finding resulted in its approval in Japan in 2015.^4^ Immune checkpoint inhibitors (ICIs) were developed in the 2010s, and the efficacy of nivolumab and pembrolizumab, both of which are anti‐programmed cell death‐1 antibodies, was evaluated across various cancers. In the ATTRACTION‐2 trial, survival was longer with nivolumab than with placebo in third‐line or later‐line treatment.^5^ This finding led to approval of nivolumab as third‐line or later‐line treatment in Japan in 2017. Pembrolizumab was also approved for cancers with high microsatellite instability as a second‐line or later‐line treatment in 2018 based on the results of KEYNOTE‐158 and KEYNOTE‐164.^6^, ^7^ Futhermore, trifluridine/tipiracil showed superiority over placebo as third‐line or later‐line therapy in the TAGS trial, leading to approval of this combination in 2019.^8^

The introduction of novel drugs into clinical practice expands the treatment options and alters treatment patterns. A Japanese administrative claims database investigated the regimens selected for each treatment line between 2014 and 2019 and found that ramucirumab‐containing therapy accounted for 72% of second‐line treatments and that nivolumab constituted 51% of third‐line treatments.^9^ Although newly approved drugs are utilized in real‐world practice, it remains unclear whether they contribute to prolonged suvival for patients in real‐world settings. Owing to the poor prognosis of gastric cancer, only a limited number of patients can receive second‐line or later‐line treatment. Approximately half of patients who receive first‐line treatment receive second‐line treatment, and about one‐quarter receive third‐line treatment.^9^ ICIs represent a promising cancer treatment, but there is a scarcity of real‐world data on the impact of ICIs on AGC. Therefore, this study aimed to elucidate the impact of ICIs on survival in patients with AGC in everyday clinical practice.

## 2 METHODS

### 2.1 Patients

The patients in this single‐institution retrospective study were diagnosed with gastric cancer between 2010 and 2020 in the Department of Gastrointestinal Medical Oncology at the National Cancer Center Hospital. The study inclusion criteria were unresectable, recurrent, or metastatic advanced gastric or esophagogastric junction (EGJ) adenocarcinoma, Eastern Cooperative Oncology Group performance status (ECOG PS) 0–2, and a history of first‐line chemotherapy with doublet or triplet regimens containing platinum. Patients who relapsed during adjuvant chemotherapy or within 6 months after completion of adjuvant chemotherapy were excluded, considering that combination therapy with fluoropyrimidine and platinum agents may be less effective in these patients.^10^, ^11^, ^12^ Patients for whom the only non‐curative resection factors was positive peritoneal cytology were also excluded because they may have a better prognosis than patients with other non‐curative resection factors.^13^

The study was approved by the Institutional Review Board of the National Cancer Center (approval number 2017‐229) and was conducted in accordance with the ethical principles outlined in the Declaration of Helsinki. The need for informed consent was waived in view of the retrospective observational nature of the research. However, patient consent was secured via the opt‐out method.

### 2.2 Assessment

The impact of ICIs on survival was assessed by dividing the patients into two groups based on whether they were treated before or after 2017, which is the year nivolumab was approved. The pre‐2017 group included patients who started treatment between 2010 and 2016 and the post‐2017 group included those who started treatment between 2017 and 2020. The primary outcome was overall survival (OS), which was defined as the time from initiation of first‐line treatment until death or was censored at the last follow‐up visit for surviving patients. The secondary outcomes included time to discontinuation (TTD) of each treatment line, survival following progression on second‐line treatment, the frequency with which each drug was used in all treatment lines and each individual treatment line, and the proportion of patients transitioning to subsequent lines of therapy. TTD was defined as the time from initiation of each treatment until the last administration or death. Patients who had not experienced treatment discontinuation or death at the time of the data cut‐off were censored.^14^ The proportion of patients transitioning to subsequent lines of therapy was calculated as the number who progressed on the previous treatment line or died divided by the number of patients who received the next treatment line. The data cut‐off date was April 30, 2021. Patient characteristics at baseline were compared using the *t*‐test, Mann–Whitney *U*‐test, chi‐squared test, or Fisher's exact test as appropriate. OS, TTD, and survival following progression of second‐line treatment were estimated using the Kaplan–Meier method and compared using the log‐rank test.

Univariate and multivariate analyses were performed with adjustment for patient background factors that might affect OS. Factors included in univariate and multivariate analyses were treatment start time (pre‐2017 vs. post‐2017), age (≥65 vs. <65 years), sex, prior gastrectomy, anatomical location (EGJ vs. gastric), histology (intestinal vs. diffuse or intestinal vs. mixed), human epidermal growth factor receptor 2 (HER2) status (positive vs. negative), ECOG PS (0–1 vs. 2), lymph node metastasis, liver metastasis, peritoneal metastasis, and lung metastasis. Patients with unknown HER2 status were excluded. All statistical analyses were performed using EZR version 1.55. A two‐tailed *p*‐value < 0.05 was considered statistically significant.

## 3 RESULTS

### 3.1 Patient Characteristics

A flow diagram showing the patient selection process is provided in Figure S1. A total of 1918 patients with gastric cancer were registered at our hospital during the study period. In total, 1093 patients were excluded for the following reasons: first‐line treatment received at another hospital (*n* = 276); relapse on adjuvant chemotherapy or within 6 months after completion of adjuvant chemotherapy (*n* = 250); advanced cancer that was unresectable, recurrent, or metastatic (*n* = 203); diagnosis other than adenocarcinoma (*n* = 194); first‐line chemotherapy that did not include a platinum agent (*n* = 140); positive peritoneal cytology as the only non‐curative resection factor (*n* = 23); and an ECOG PS of 3 (*n* = 7). Finally, 825 eligible patients were included in the study (pre‐2017 group, *n* = 533; post‐2017 group, *n* = 292).

Patient characteristics at the start of first‐line treatment are presented in Table 1. The median age in the pre‐2017 and the post‐2017 groups were 64 and 65 years, respectively, and males constituted 373 (70.0%) and 192 (65.8%) of these groups, respectively. Patients in the pre‐2017 group tended to have better ECOG PS, be less likely to have the EGJ as the primary site, and be more likely to have liver metastasis. Unknown HER2 status was more common in the pre‐2017 group because trastuzumab was not approved in 2010 and HER2 testing was not performed.

**TABLE 1:** Patient characteristics at the start of first‐line treatment.

	Pre‐2017 *n* (%)	Post‐2017 *n* (%)	*p*‐value
Total number	533	292	
Age, years			
median [range]	64 [20–83]	65 [25–85]	0.63
Sex, male	373 (70.0)	192 (65.8)	0.21
ECOG PS			
0	206 (38.6)	89 (30.6)	0.06
1	297 (55.7)	182 (62.3)	
2	30 (5.6)	21 (7.2)	
Histology			
Intestinal	126 (23.6)	64 (21.9)	0.37
Diffuse	276 (51.8)	143 (49.0)	
Mixed	131 (24.6)	85 (29.1)	
HER2 status			
Positive	82 (15.4)	57 (19.5)	<0.01
Negative	366 (68.7)	230 (78.8)	
Unknown	85 (15.9)	5 (1.7)	
Prior gastrectomy			
No	419 (78.6)	229 (78.4)	1
Yes	114 (21.4)	63 (21.6)	
Primary site			
Gastric	501 (94.0)	264 (90.4)	0.07
EGJ	32 (6.0)	28 (9.6)	
Site of metastasis			
Lymph node	398 (74.7)	224 (77.0)	0.60
Peritoneum	334 (62.7)	189 (64.9)	0.55
Liver	157 (29.5)	69 (23.6)	0.09
Lung	39 (7.3)	21 (7.2)	1

Abbreviations: ECOG PS, Eastern Cooperative Oncology Group performance status; EGJ, esophagogastric junction; HER2, human epidermal growth factor receptor 2.

### 3.2 Overall survival

**FIGURE 1:**
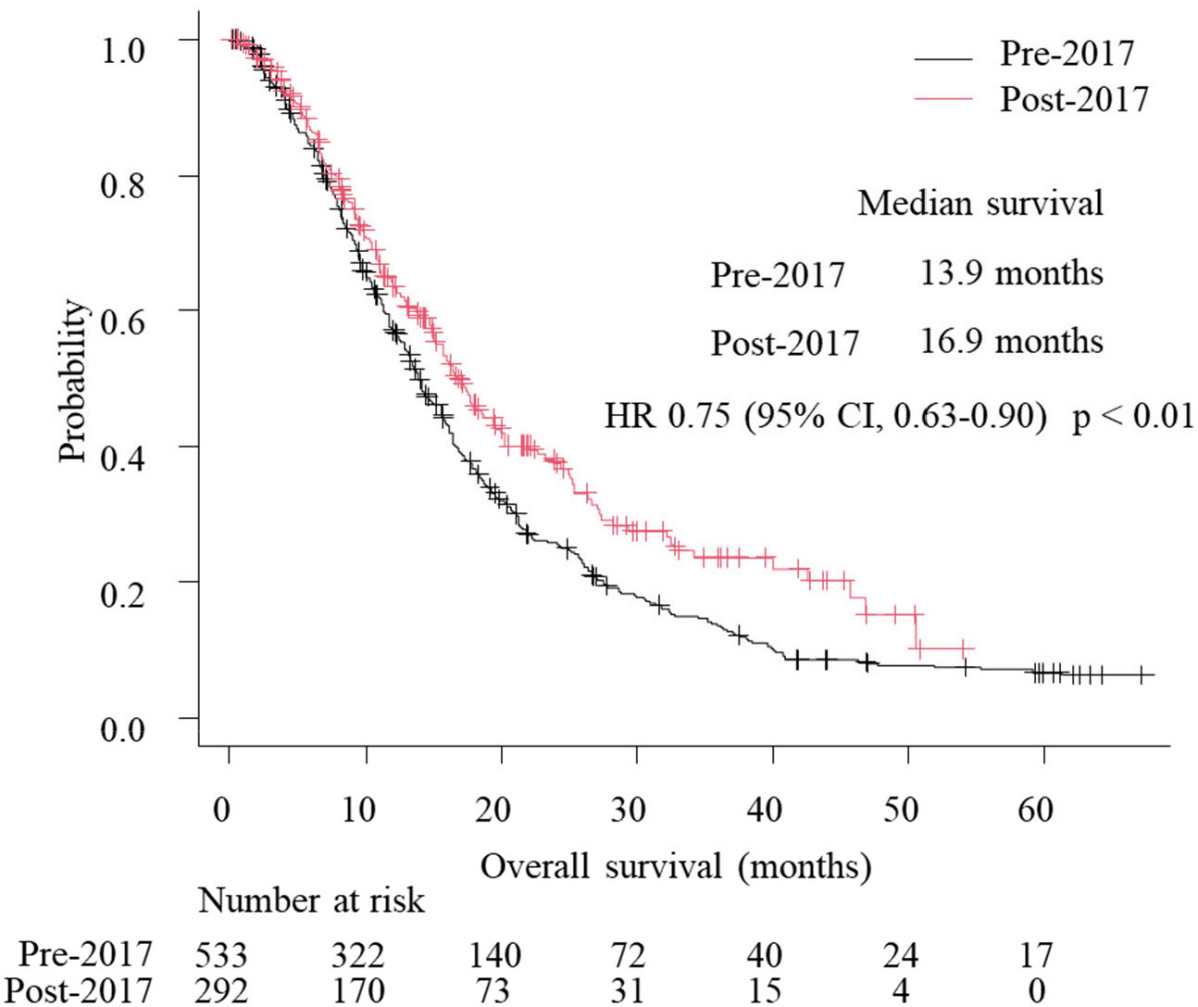
Kaplan–Meier curves showing overall survival, HR, hazard ratio; CI, confidential interval.

The median follow‐up duration was 12.2 months. Patients in the post‐2017 group had a significantly longer median OS than those in the pre‐2017 group (16.9 vs. 13.9 months; hazard ratio [HR] 0.75, 95% confidence interval [CI], 0.63–0.90; *p* < 0.01) (Figure 1). Ninety patients with unknown HER2 status were excluded from the univariate and multivariate analyses. In univariate analysis, post‐2017 treatment (HR 0.75, 95% CI 0.63–0.90; *p* < 0.01), recurrence (HR 0.73, 95% CI 0.60–0.89; *p* < 0.01), and HER2‐positive status (HR 0.75, 95% CI 0.60–0.95; *p* = 0.02) were significantly related to a favorable prognosis, while diffuse type (HR 1.38, 95% CI 1.18–1.62; *p* < 0.01), an ECOG PS of 2 (HR 2.51, 95% CI 1.82–3.46; *p* < 0.01), liver metastasis (HR 1.20, 95% CI 1.01–1.44; *p* = 0.04), and peritoneal metastasis (HR 1.74, 95% CI 1.47–2.07; *p* < 0.01) were significantly related to a poor prognosis. Multivariate analysis revealed that post‐2017 treatment (HR 0.79, 95% CI 0.66–0.96; *p* = 0.02) and HER2‐positive status (HR 0.75, 95% CI 0.58–0.97; *p* = 0.03) were significantly favorable prognostic factors while diffuse type (HR 1.53, 95% CI 1.19–1.96; *p* < 0.01), an ECOG of PS 2 (HR 2.52, 95% CI 1.71–3.70; *p* < 0.01), lymph node metastasis (HR 1.39, 95% CI 1.09–1.76; *p* < 0.01), liver metastasis (HR 1.59, 95% CI 1.28–1.98; *p* < 0.01), and peritoneal metastasis (HR 1.94, 95% CI 1.56–2.40; *p* < 0.01) were significantly poor prognostic factors (Table 2). A sensitivity analysis that only includes factors that were *p* < 0.05 in the univariate analysis resulted in similar findings (Table S1).

**TABLE 2:** Univariate and multivariate analysis of overall survival.

Covariate	Univariate		Multivariate	
	HR (95% CI)	*p*‐values	HR (95% CI)	*p*‐values
Start of treatment				
Post‐2017 (vs. Pre‐2017)	0.75 (0.63–0.90)	<0.01	0.79 (0.66–0.96)	0.02
Age				
≥65 years (vs. <65 years)	0.94 (0.80–1.11)	0.46	0.92 (0.77–1.10)	0.37
Sex				
Female (vs. Male)	0.99 (0.84–1.18)	0.95	0.99 (0.81–1.21)	0.93
Prior gastrectomy				
Yes (vs. No)	0.73 (0.60–0.89)	<0.01	0.85 (0.67–1.09)	0.20
Location				
EGJ (vs. Gastric)	0.80 (0.59–1.10)	0.17	1.21 (0.86–1.69)	0.28
Histology (vs. Intestinal)				
Diffuse	1.38 (1.18–1.62)	<0.01	1.53 (1.19–1.96)	<0.01
Mixed	0.94 (0.78–1.13)	0.52	1.22 (0.94–1.58)	0.14
HER2				
Positive (vs. Negative)	0.75 (0.60–0.95)	0.02	0.75 (0.58–0.97)	0.03
ECOG PS				
2 (vs. 0–1)	2.51 (1.82–3.46)	<0.01	2.52 (1.71–3.70)	<0.01
Lymph node metastasis				
Yes (vs. No)	1.05 (0.87–1.26)	0.63	1.39 (1.09–1.76)	<0.01
Liver metastasis				
Yes (vs. No)	1.20 (1.01–1.44)	0.04	1.59 (1.28–1.98)	<0.01
Peritoneal metastasis				
Yes (vs. No)	1.74 (1.47–2.07)	<0.01	1.94 (1.56–2.40)	<0.01
Lung metastasis				
Yes (vs. No)	0.99 (0.72–1.36)	0.94	1.08 (0.76–1.55)	0.66

Abbreviations: CI, confidence interval; ECOG PS, Eastern Cooperative Oncology Group Performance Status; EGJ, esophagogastric junction; HER2, human epidermal growth factor receptor 2; HR, hazard ratio.

**FIGURE 2:**
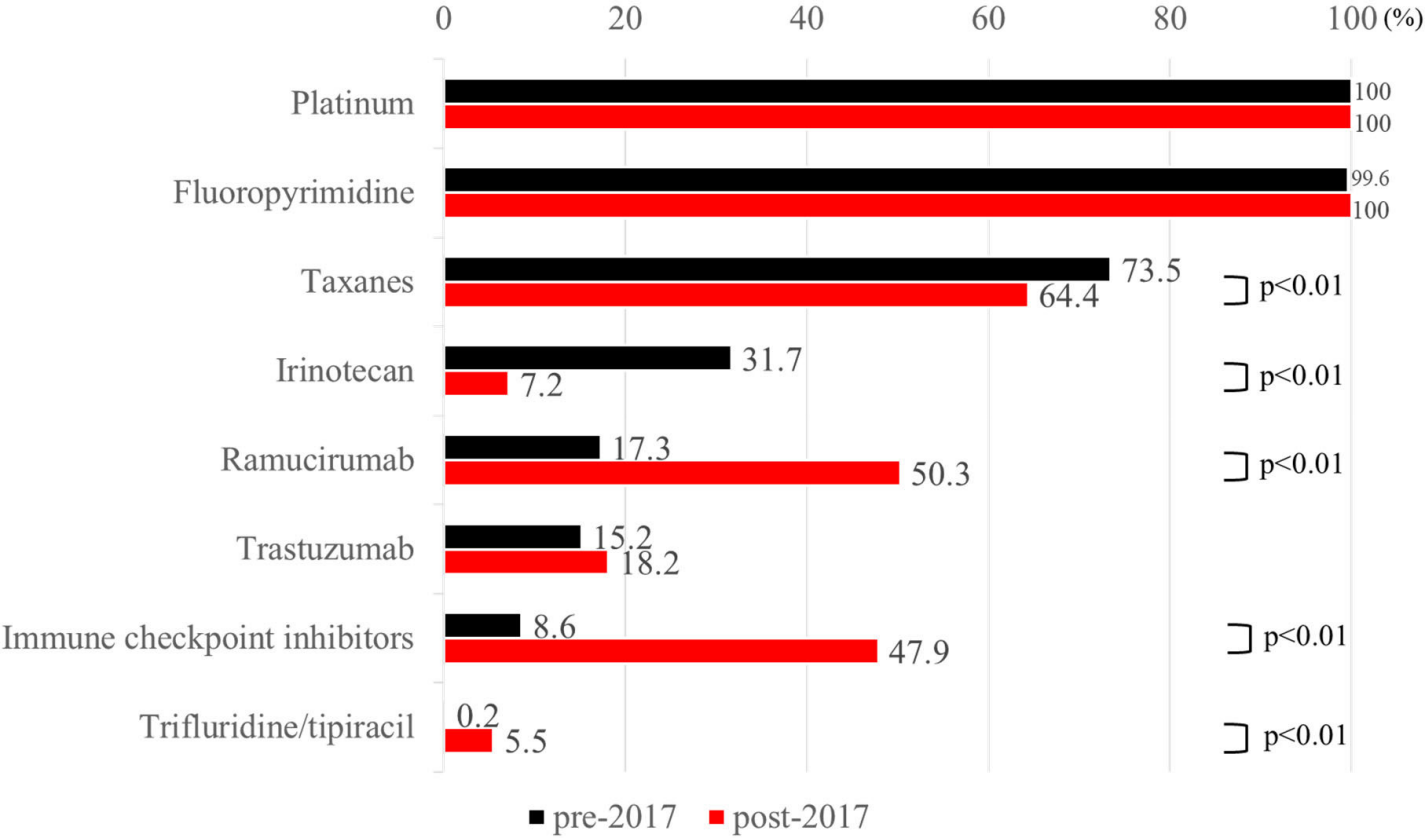
Percentage of each drug used in all treatment lines.

The frequency with which each drug was used in all treatment lines is shown in Figure 2. Only one patient who received platinum and irinotecan combination therapy as first‐line treatment did not receive fluoropyrimidine. Taxanes (73.5% vs. 64.4%, *p* < 0.01) and irinotecan (31.7% vs. 7.2%, *p* < 0.01) were used significantly more often in the pre‐2017 group than in the post‐2017 group, while ramucirumab (17.3% vs. 50.3%, *p* < 0.01), ICIs (8.6% vs. 47.9%, *p* < 0.01), and trifluridine/tipiracil (0.2% vs. 5.5%, *p* < 0.01) were used significantly more often in the post‐2017 group.

### 3.3 TTD for each treatment line

In first‐line treatment, there was no significant difference in median TTD between the pre‐2017 group and the post‐2017 group (6.0 vs. 5.9 months; HR 0.98, 95% CI 0.84–1.14; *p* = 0.79) (Figure S2). Most patients in both groups received a fluoropyrimidine plus platinum regimen (71.1% vs. 67.5%). A triplet regimen consisting of fluoropyrimidine, platinum, and taxane was used more often in the pre‐2017 group than in the post‐2017 group (15.2% vs. 0.7%), while a fluoropyrimidine, platinum, and ICI regimen was used more often in the post‐2017 group (1.3% vs. 10.3%) (Figure S3).

In the pre‐2017 and post‐2017 groups, 403 and 197 patients, respectively, received second‐line treatment. There was no significant difference in the median TTD for second‐line treatment between the pre‐2017 group and the post‐2017 group (3.0 vs. 3.1 months; HR 0.95, 95% CI 0.79–1.13; *p* = 0.67) (Figure S4). In patients who received second‐line treatment, taxane monotherapy (44.2% vs. 19.8%) and irinotecan monotherapy (10.4% vs. 1.0%) were used more frequently in the pre‐2017 group than in the post‐2017 group, while taxane plus ramucirumab therapy was used more frequently as second‐line treatment in the post‐2017 group (13.4% vs. 65.0%) (Figure S5).

Third‐line treatment was administered to 203 patients in the pre‐2017 group and 130 in the post‐2017 group; there was no significant difference in the median TTD for third‐line treatment between the two groups (2.1 vs. 1.8 months; HR 1.06, 95% CI 0.84–1.33; *p* = 0.62) (Figure S6). In patients who received third‐line treatment, irinotecan monotherapy (40.4% vs. 3.8%), taxane monotherapy (26.6% vs. 7.7%), and fluoropyrimidine plus platinum therapy (5.9% vs. 1.5%) were administered more frequently in the pre‐2017 group than in the post‐2017 group, while ICIs (5.9% vs. 62.3%) and trifluridine/tipiracil (0% vs. 4.6%) were administered more frequently in the post‐2017 group (Figure S7).

Seventy‐four patients in the pre‐2017 group and 47 in the post‐2017 group received fourth‐line treatment; the median TTD for fourth‐line treatment was comparable between the two groups (2.1 vs. 1.8 months; HR 0.84, 95% CI 0.57–1.25; *p* = 0.34) (Figure S8). Fluoropyrimidine plus platinum agents (24.3% vs. 14.9%), ICIs (16.2% vs. 27.7%), taxanes (13.5% vs. 4.3%), and irinotecan (12.2% vs. 27.7%) were used as fourth‐line treatment in the pre‐2017 and post‐2017 groups, respectively (Figure S9).

### 3.4 Proportion of patients transitioning to subsequent lines of therapy and survival following progression on second‐line treatment

**FIGURE 3:**
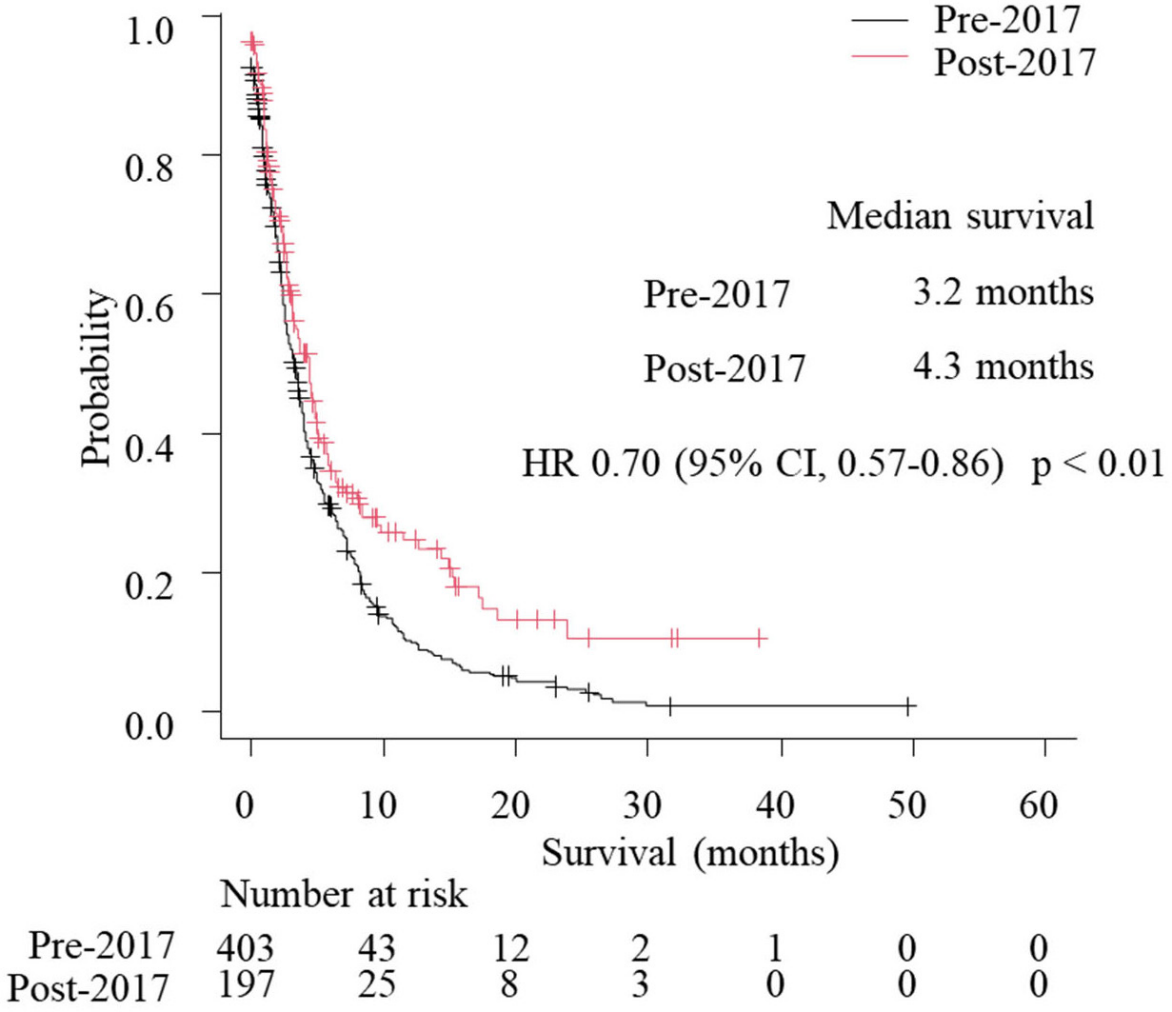
Kaplan–Meier curves showing survival following second‐line progression, HR, hazard ratio; CI, confidential interval.

**FIGURE 4:**
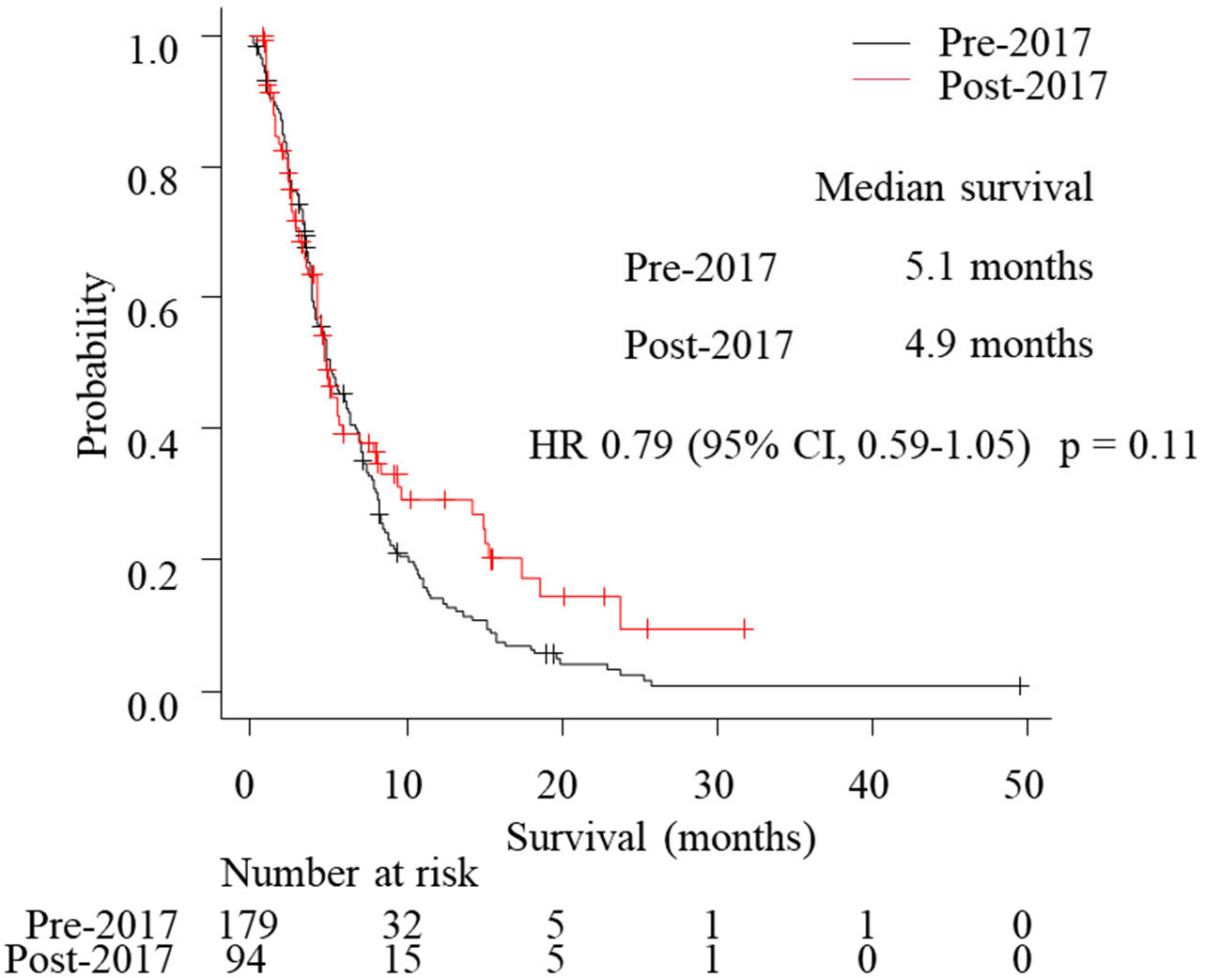
Kaplan–Meier curves comparing survival from initiation of third‐line treatment between patients received third‐line treatment other than ICIs in the pre‐2017 group and patients received ICIs as third‐line or later‐line treatment in the post‐2017 group.

The numbers of patients who experienced progression after first‐line treatment were 489 in the pre‐2017 group and 250 in the post‐2017 group. Among them, 403 and 197 patients received second‐line treatment and 278 and 178 patients experienced progression. Of 203 and 130 patients who received third‐line treatment, 195 and 116 patients had progression. Afterwards, 74 and 47 patients received fourth‐line treatment. The proportions of patients transitioning to second‐line, third‐line, and fourth‐line treatment were, respectively, 82.4% in the pre‐2017 group and 78.8% in the post‐2017 group (*p* = 0.23), 43.8% and 56.3% (*p* < 0.01), and 16.3% and 21.7% (*p* = 0.11) (Figure S10). Median survival following second‐line progression was significantly longer in the post‐2017 group than in the pre‐2017 group (4.3 vs. 3.2 months; HR 0.70, 95% CI 0.57–0.86; *p* < 0.01) (Figure 3). To explore the impact of ICIs on survival, the survival from the initiation of third‐line treatment was compared between patients who received third‐line treatment other than ICIs in the pre‐2017 group and patients who received ICIs as third‐line or later‐line treatment in the post‐2017 group. Patients who received ICIs as third‐line or later‐line treatment in the post‐2017 group had a trend of better survival than patients who received third‐line treatment other than ICIs in the pre‐2017 group (4.9 vs. 5.1 months; HR 0.79, 95% CI 0.59–1.05; *p* = 0.11) (Figure 4).

## 4 DISCUSSION

Some trials have shown the efficacy of ICIs plus chemotherapy after 2020.^15^, ^16^, ^17^ Because patients diagnosed with gastric cancer between 2010 and 2020 were eligible in this study, almost all patients received ICIs as third‐line or later‐line treatment. This study found a decrease in the use of taxanes and irinotecan in the post‐2017 group when compared with the pre‐2017 group and an increase in the use of newly approved drugs, including ramucirumab, ICIs, and trifluridine/tipiracil. OS was significantly longer in the post‐2017 group than in the pre‐2017 group. Moreover, a multivariate analysis conducted to account for imbalances in patient demographics indicated that initiating treatment after 2017 was associated with a favorable prognosis. The number of patients transitioning to third‐line treatments was significantly higher in the post‐2017 group than in the pre‐2017 group, and survival was significantly longer following progression on second‐line therapy in the post‐2017 group.

Real‐world data consist of information on patient populations selected by physicians for treatment, the treatment options, methods of administration, treatment outcomes, adverse effects, and survival rates. These data reflect a broader patient population and the actual use and side effects of therapies. Therefore, unlike clinical trials, they provide a more accurate picture of treatment outcomes under a variety of circumstances. Furthermore, real‐world data are considered to have higher general reliability in terms of treatment outcomes than clinical trials because they represent actual use in real‐world clinical settings. By considering real‐world data in conjunction with clinical trial data, more accurate information on treatment outcomes can be gleaned. Several previous studies have used real‐world data to investigate changes in the prognosis of patients with AGC. A population‐based study conducted in Europe between 1999 and 2007 showed a slight improvement in the survival rate of patients with gastric cancer in 2005–2007 in comparison with that in 1999–2001.^18^ Similarly, a National Cancer Incidence Database study in Korea revealed that patients with gastric cancer had significantly better survival rates in 2010–2014 than in 1993–1995.^19^ These studies included patients with gastric cancer that was not only advanced but also at the local and locoregional stages. A registry‐based study in the US examined survival of patients with gastric cancer by stage between 2001 and 2009 and found longer survival in patients with AGC in 2004–2009 than in those with AGC in 2001–2003.^20^ In Japan, a regional population‐based cancer registry study that included data for 1995–2018 showed a trend of improvement in the survival rate for patients with AGC over time.^21^ Furthermore, a single‐institution retrospective study that included data for 2007–2018 found that OS in patients with AGC was prolonged across different time periods when adjusted for prognostic factors.^22^ Few patients in these studies received nivolumab because it was not approved in Japan until 2017.

Cytotoxic agents for advanced gastric cancer were developed before 2010, but molecular targeted agents did not emerge until after 2010. ICIs in particular have shown promising efficacy and the potential for a long‐term response. The ATTRACTION‐2 trial, which examined the efficacy of nivolumab therapy after second‐line treatment for patients with AGC, reported 2‐ and 3‐year survival rates of 10.6% and 5.6%, respectively.^23^ Our study mainly evaluated the real‐world clinical impact of ICIs by dividing patients into pre‐2017 and post‐2017 groups because nivolumab was approved as third‐line or later‐line treatment in 2017. In the pre‐2017 group, 46 patients (8.6%) received ICIs while 140 (47.9%) in the post‐2017 group received these agents. The majority of these patients received ICIs as third‐line therapy. Patients received ICIs as third‐line or later‐line treatment in the post‐2017 group showed longer survival than patients received third‐line treatment other than ICIs in the pre‐2017 group. Meanwhile, no differences in TTD were observed between the two groups and no long‐term responses noted. This could be attributable to the following reasons: unlike in ATTRACTION‐2, our study did not compare ICIs with placebo; TTD may have excluded patients who discontinued treatment because of adverse events but survived for a long period of time; and the follow‐up duration was limited. Our finding that OS improved significantly after approval of ICIs could be attributed to an increased proportion of patients transitioning to third‐line and fourth‐line treatments and prolonged survival following progression on second‐line treatment. Three or more lines of sequential treatment are known to contribute to longer survival. A systematic review and meta‐analysis of Phase III studies of chemotherapy for AGC showed that third‐line treatment prolonged both OS and post‐progression survival.^24^ Furthermore, a retrospective study from the UK reported a substantial correlation between survival and number of treatment lines received.^25^ The proportion of patients receiving third‐line or later‐line treatment may have increased because of introduction of newly approved drugs, which have expanded the number of treatment options.

Despite the large cohort of over 800 patients in this study, many patient background factors, as well as regimens and survival times on each line of treatment were provided. There were few missing patient background factors, and a multivariate analysis provided reliable results, including known prognostic factors. Considering the limited real‐world data available on ICIs for AGC, this study is valuable because it provides information on the efficacy of ICIs in clinical practice. Moreover, this study has supported the importance of salvage‐line treatment. Since ICIs have been used as first‐line treatment for AGC now, the change in salvage‐line treatment and survival outcomes in real‐world practice needs to be evaluated.

However, our study had several limitations. First, we assessed TTD rather than progression‐free survival (PFS). Although PFS is a common endpoint for evaluating the efficacy of anticancer drugs, measuring PFS in the real‐world requires manual image assessment because PFS is rarely measured by RECIST in routine clinical practice. However, TTD is easy to measure even in the real‐world setting but does not necessarily define tumor progression as an event. In the absence of tumor progression, discontinuation of treatment owing to adverse events, the patient's request, or the planned number of cycles is counted as an event, and TTD may underestimate true PFS. Conversely, if treatment continues despite disease progression, TTD may overestimate true PFS. There are limitations in using TTD as an endpoint, but it has been reported that TTD is correlated with PFS and has the potential to be a surrogate endpoint.^14^ Second, significantly more triplet regimens consisting of fluoropyrimidine, platinum, and taxane was used in first‐line treatment, which may have affected the proportion of patients shifted to third‐line or fourth‐line treatment. The increase in triplet therapy has likely been influenced by clinical trials conducted in Japan comparing docetaxel, cisplatin, and S‐1 with cisplatin and S‐1 therapy in the early 2010s. PFS and OS did not differ between these two groups.^26^ Third, laboratory test data were not collected. Alkaline phosphatase, asparate transaminase, albumin, total bilirubin, lactate dehydrogenase, and the neutrophil‐to‐lymphocyte ratio have been reported to be associated with the prognosis in patients with AGC.^27^ Fourth, the follow‐up duration was short. Because the data cutoff was April 2021, patients in the post‐2017 group experienced more censoring than those in the pre‐2017 group. Fifth, improvements in supportive care or lead‐time biases such as earlier diagnosis may have affected survival.

In conclusion, approval of nivolumab for use in clinical practice might have been associated with an increased rate of transition to third‐line treatment and prolonged survival after progression on second‐line therapy. These therapeutic advances could contribute to extended OS in patients with AGC in real‐world practice.

## AUTHOR CONTRIBUTIONS

**Toru Kadono:** Conceptualization (equal); data curation (lead); formal analysis (lead); writing – original draft (lead); writing – review and editing (equal). **Satoru Iwasa:** Conceptualization (equal); writing – review and editing (equal). **Toshiharu Hirose:** Writing – review and editing (equal). **Hidekazu Hirano:** Writing – review and editing (equal). **Natsuko Okita:** Writing – review and editing (equal). **Hirokazu Shoji:** Writing – review and editing (equal). **Atsuo Takashima:** Writing – review and editing (equal). **Ken Kato:** Writing – review and editing (equal).

## FUNDING INFORMATION

This research did not receive any specific grants from funding agencies in the public, commercial, or not‐for‐profit sectors.

## CONFLICT OF INTEREST STATEMENT

Author Satoru Iwasa serves to CHUGAI PHARMACEUTICAL CO., LTD. Author Hidekazu Hirano has received research grants from Beigene, TAIHO, Seagen, Amgen, ALX Oncology and Bristol‐Myers Squibb and a speaker honorarium from Bristol‐Myers Squibb, TAIHO, Novartis, NICHI‐IKO, Teijin Pharma and Ono Pharmaceutical. Author Hirokazu Shoji has received research grants from TAIHO, Amgen, Astellas, MSD, Takeda, Daiichisankyo and Ono Pharmaceutical and a speaker honorarium from Bristol‐Myers Squibb, Astellas, MSD and Abbvie. Author Atsuo Takashima has received research grants from MSD, Daiichisankyo, Pfizer, Hutchison MediPharma, Incyte, Isofol Medical AB, Eisai and Ono Pharmaceutical and a speaker honorarium from TAIHO, Takeda, Lilly, Ono Pharmaceutical, Chugai‐pharm, Merck and Bayer. Author Ken Kato has received research grants from TAIHO, Astellas, MSD, Daiichisankyo, Beigene, Chugai‐pharm, Bayer, Janssen, Merck, MSD, Astrazeneca and Ono Pharmaceutical and a speaker honorarium from Bristol‐Myers Squibb, MSD, Lilly, Chugai‐pharm, Takeda, Merck, Ono Pharmaceutical, Bayer, TAIHO, Tsumura, Otsuka Pharmaceutical, Daiichi Sankyo, Seagen, Oncolys Bio Pharma and Beigene. The other authors have nothing to disclose.

## PRECIS

Patients with advanced gastric cancer had significantly longer survival after approval of nivolumab. Survival outcomes after progression on second‐line treatment were longer after approval of nivolumab.

## Supporting information


Figure S1.

Table S1.

## Data Availability

The datasets generated during the current study are not publicly available due to ethical restrictions but are available from the corresponding author upon reasonable request.
