# Supplementary material for: Impact of immune checkpoint inhibitors on survival outcomes in advanced gastric cancer in Japan: A real‐world analysis
Source: Cancer Med. 2024 Jun 20;13(12):e7401. doi: 10.1002/cam4.7401 (PMC11187802; doi:10.1002/cam4.7401)

**Appendix Figure Legends**

Figure A1. Patient flow diagram. ECOG PS, Eastern Cooperative Oncology Group Performance Status

Figure A2. Kaplan–Meier curves showing time-to-treatment-discontinuation in first-line treatment

Figure A3. Proportion of regimens in first-line treatment. FP, fluoropyrimidine; Tmab, trastuzumab; ICI, immune checkpoint inhibitor

Figure A4. Kaplan–Meier curves showing time-to-treatment-discontinuation in second-line treatment

Figure A5. Proportion of regimens in second-line treatment. RAM, ramucirumab; FP, fluoropyrimidine; Tmab, trastuzumab

Figure A6. Kaplan–Meier curves showing time-to-treatment-discontinuation in third-line treatment

Figure A7. Proportion of regimens in third-line treatment. RAM, ramucirumab; ICI, immune checkpoint inhibitor; FP, fluoropyrimidine; FTD/TPI, trifluridine/tipiracil

Figure A8. Kaplan–Meier curves showing time-to-treatment-discontinuation in fourth-line treatment

Figure A9. Proportion of regimens in third-line treatment. FP, fluoropyrimidine; ICI, immune checkpoint inhibitor

Figure A10. Proportion of patient transitioning to subsequent lines of therapy

Figure A1.


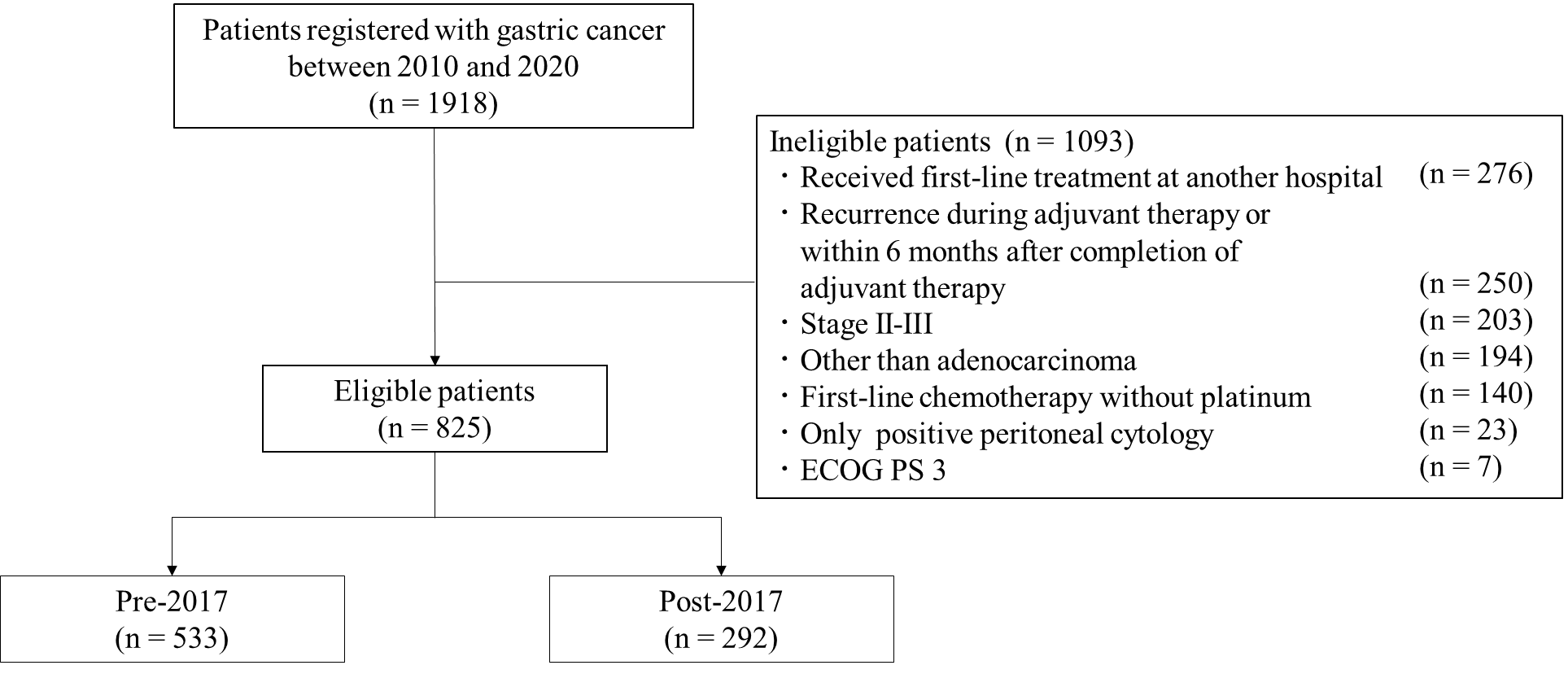


Figure A2.


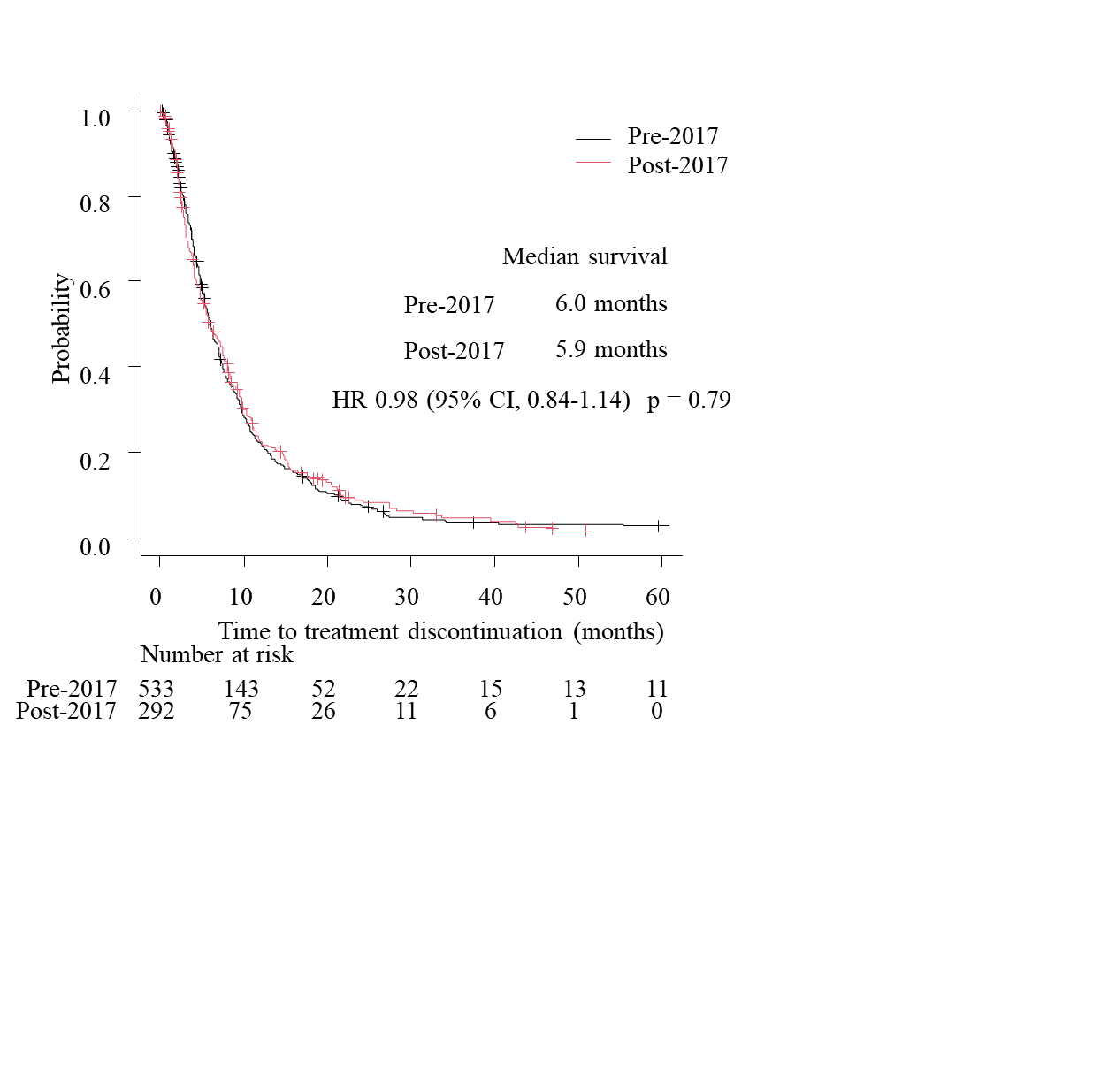


Figure A3.


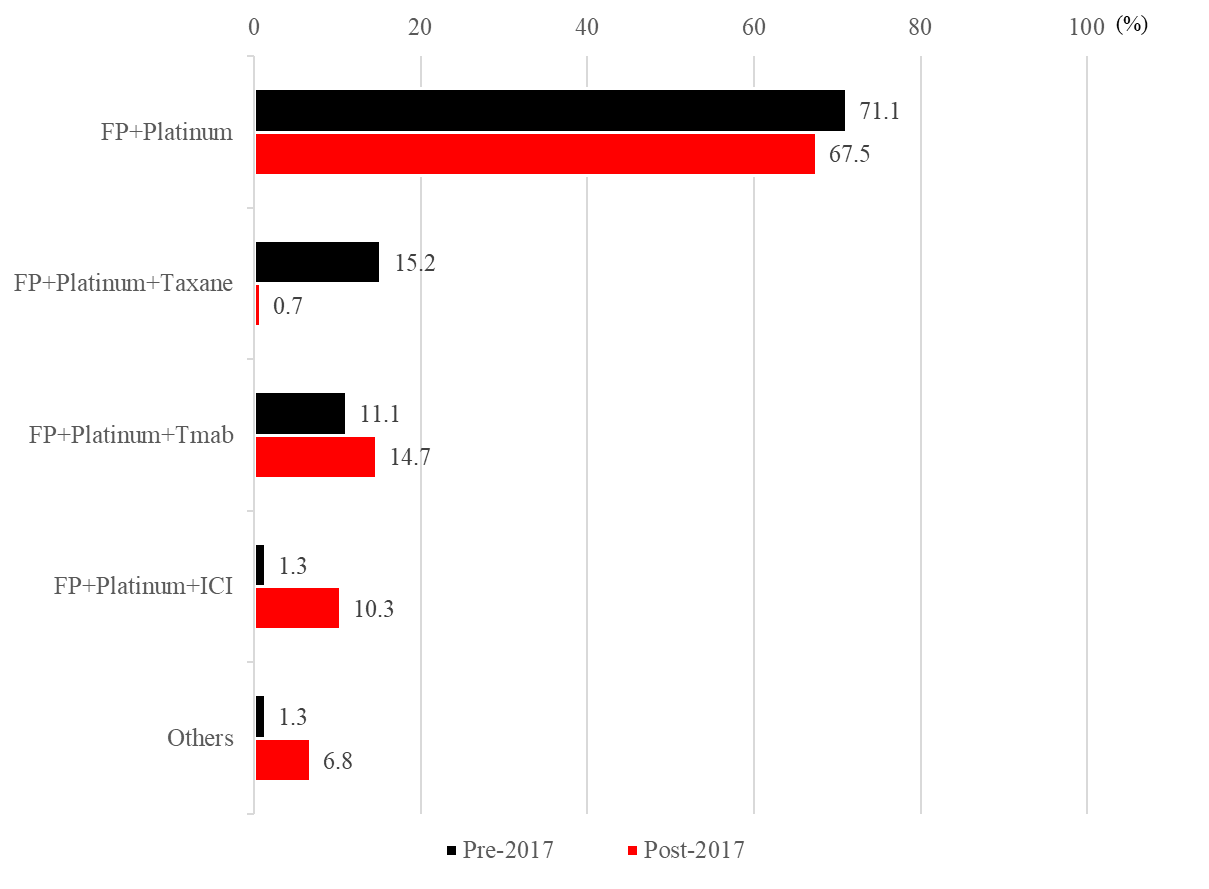


Figure A4.


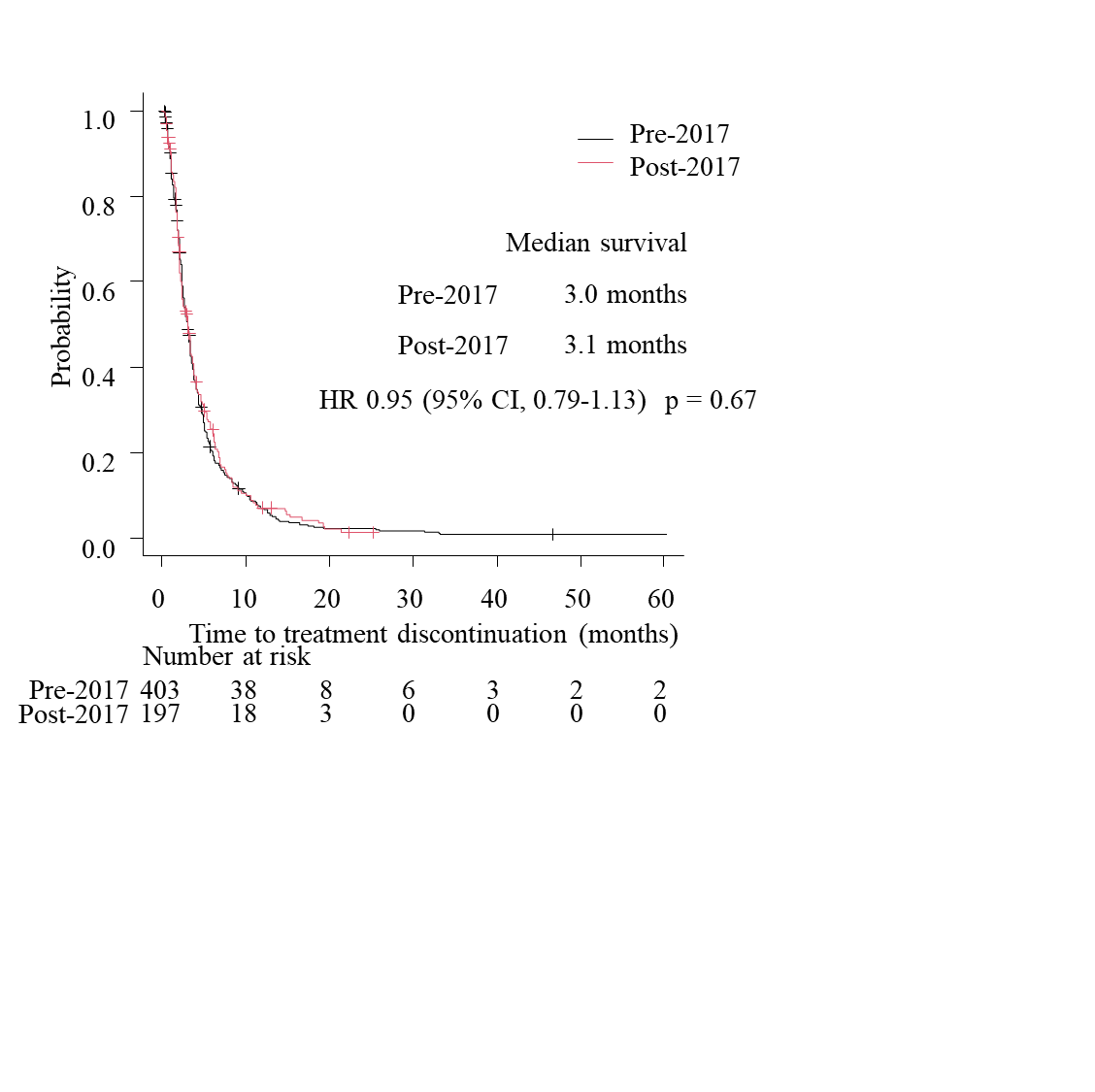


Figure A5.


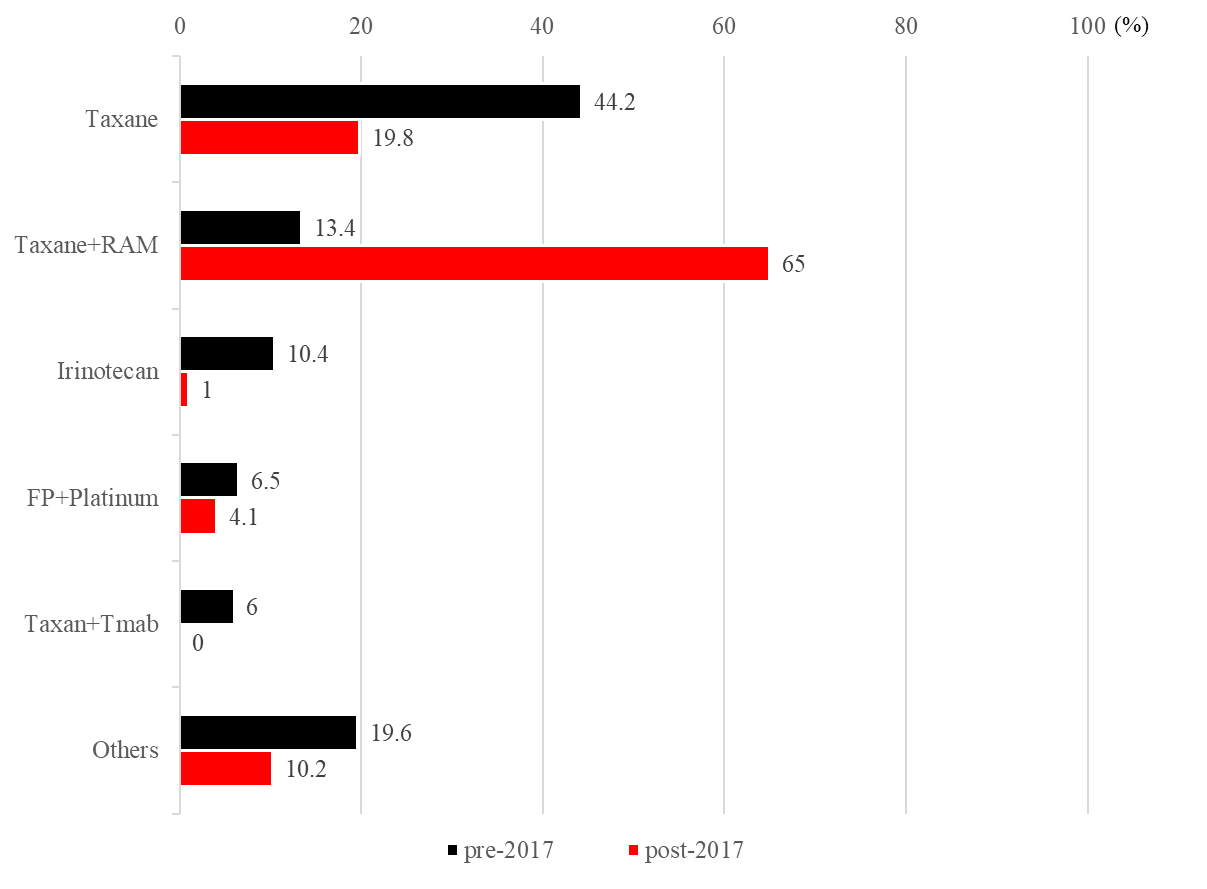


Figure A6.


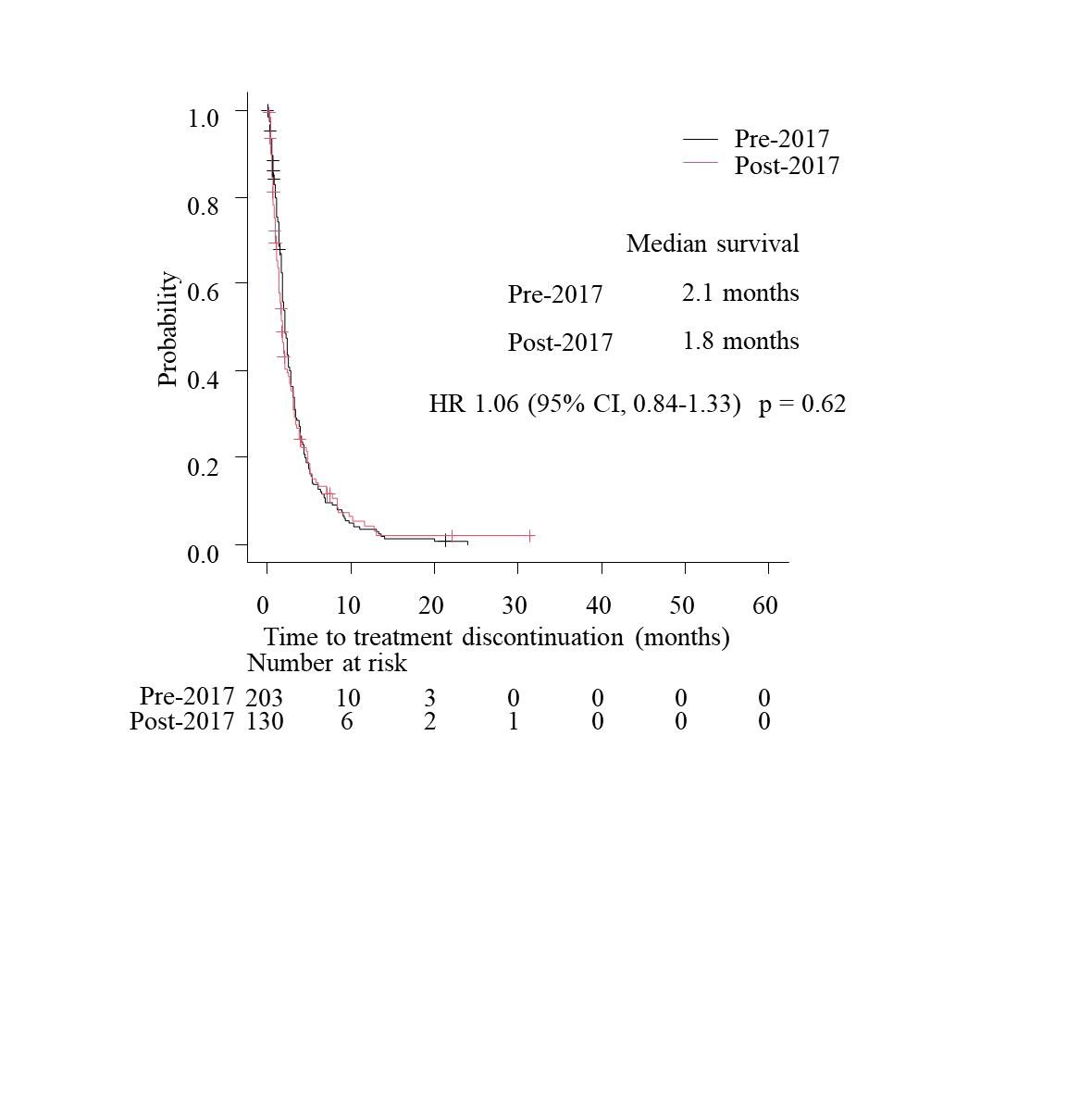


Figure A7.


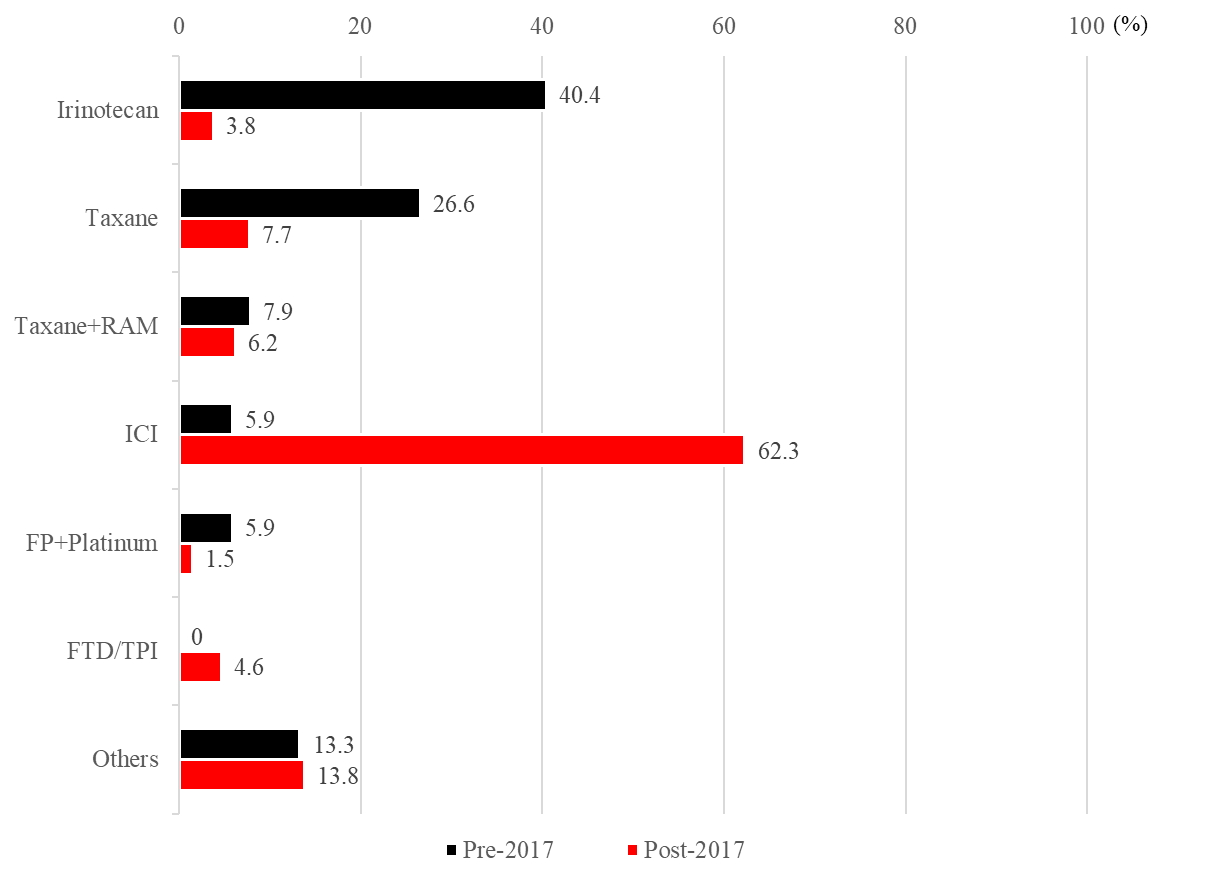


Figure A8.


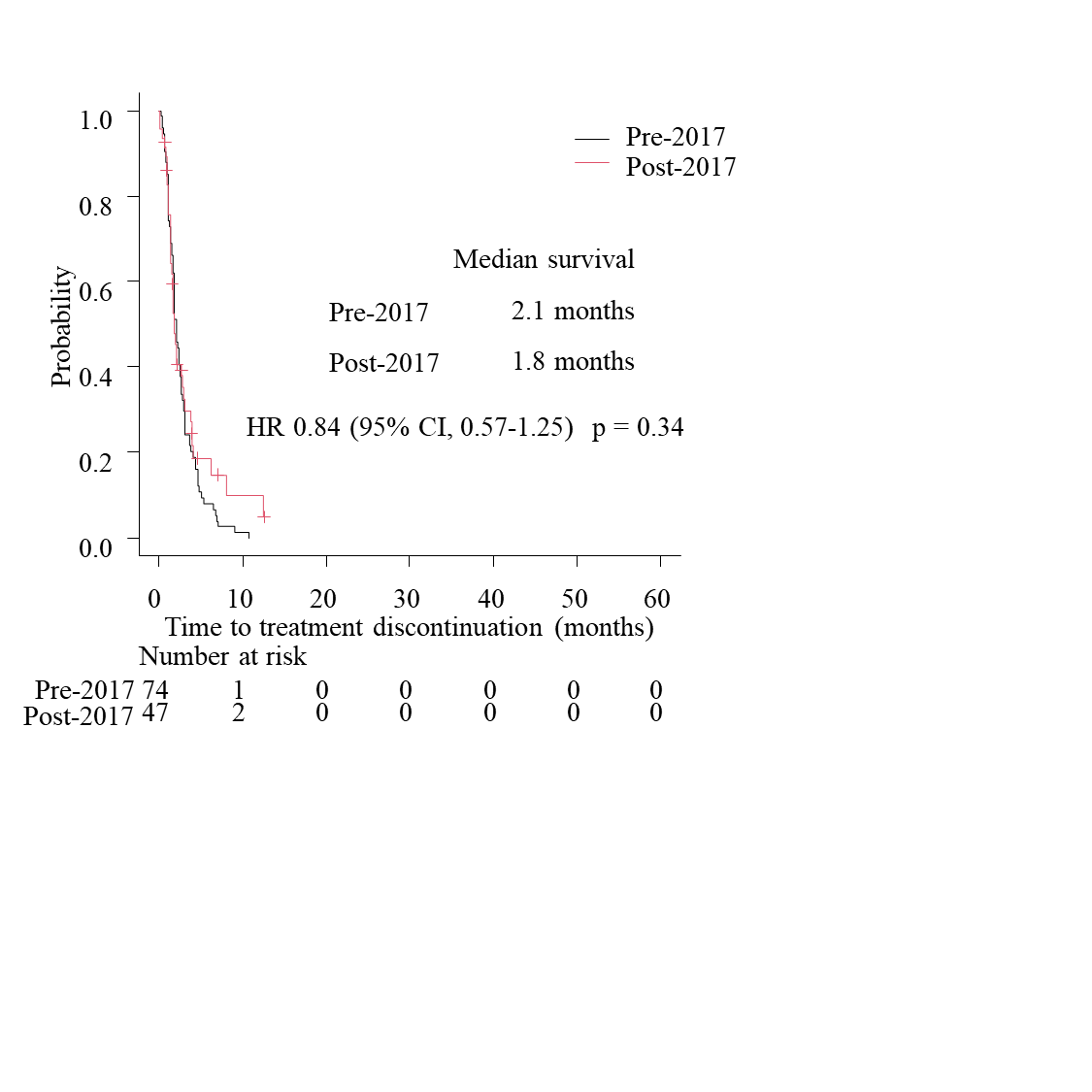


Figure A9.


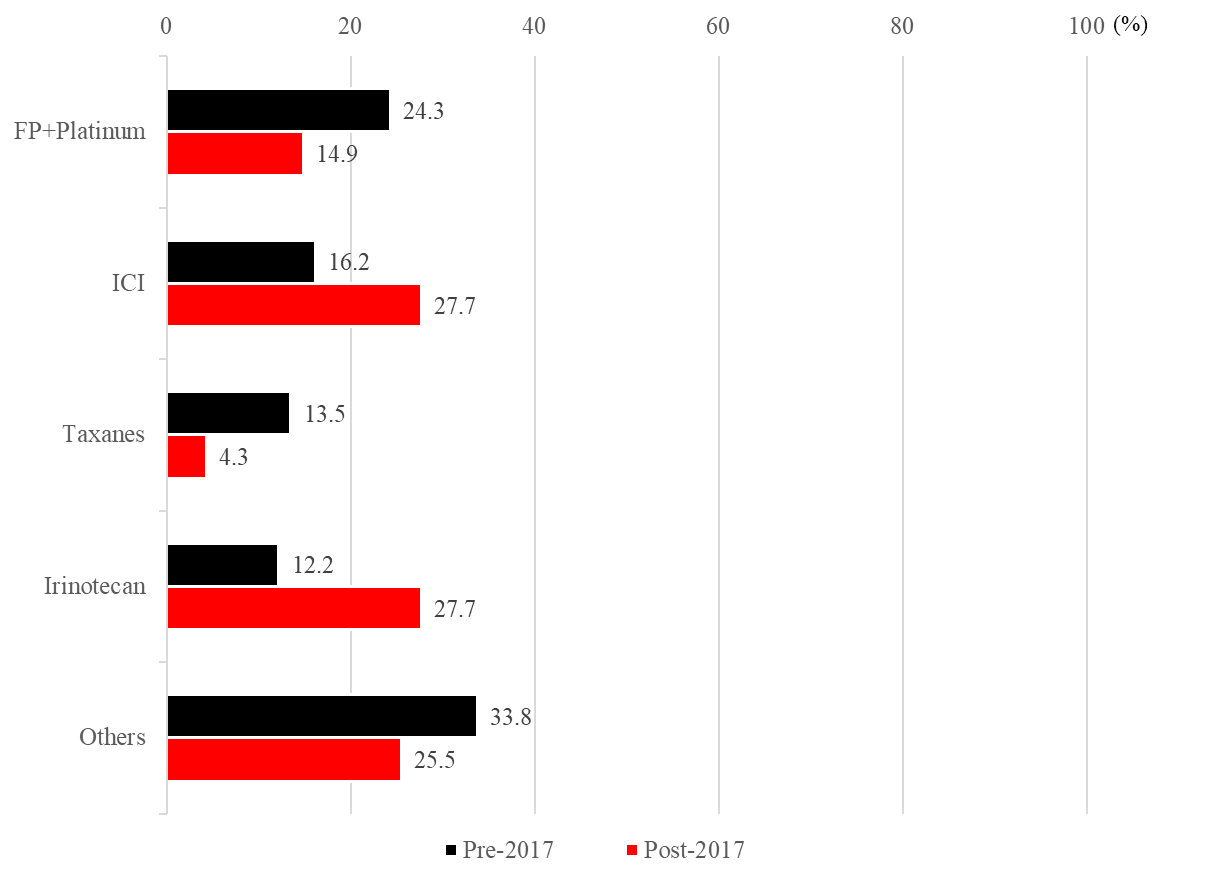


Figure A10.

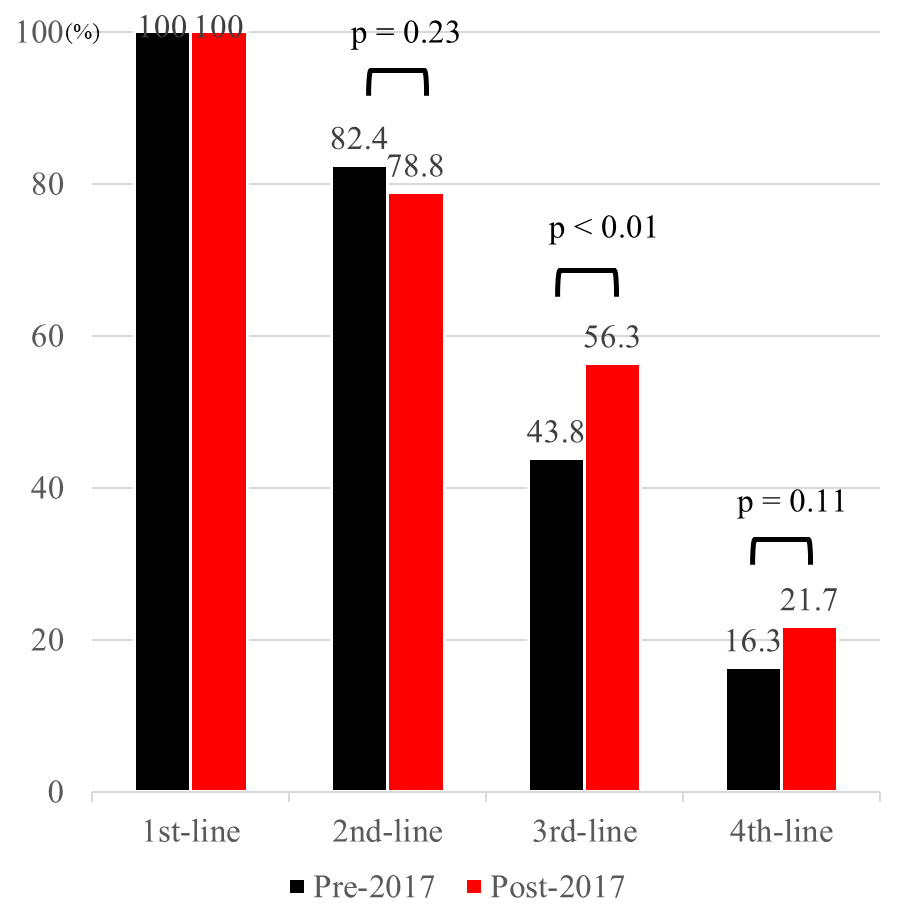

Supplement: Supplementary file 1 — Figure S1. [file CAM4-13-e7401-s001.docx]
