# Supplementary material for: Impact of immune checkpoint inhibitors on survival outcomes in advanced gastric cancer in Japan: A real‐world analysis
Source: Cancer Med. 2024 Jun 20;13(12):e7401. doi: 10.1002/cam4.7401 (PMC11187802; doi:10.1002/cam4.7401)
Supplement: Supplementary file 2 — Table S1. [file CAM4-13-e7401-s002.docx]

Table S1. Sensitivity analysis of overall survival

|  |  | Multivariate | |
| --- | --- | --- | --- |
| Covariate |  | HR (95% CI) | p-value |
| Start of treatment |  |  |  |
| Post-2017 (vs. Pre-2017) |  | 0.79 (0.66–0.96) | 0.02 |
| Prior gastrectomy |  |  |  |
| Yes (vs. No) |  | 0.76 (0.61–0.94) | 0.01 |
| Histology (vs. Intestinal) |  |  |  |
| Diffuse |  | 1.48 (1.16–1.88) | < 0.01 |
| Mixed |  | 1.26 (0.97–1.63) | 0.09 |
| HER2 |  |  |  |
| Positive (vs. Negative) |  | 0.79 (0.62–1.01) | 0.06 |
| ECOG PS |  |  |  |
| 2 (vs. 0-1) |  | 2.27 (1.56–3.29) | < 0.01 |
| Liver metastasis |  |  |  |
| Yes (vs. No) |  | 1.57 (1.26–1.96) | < 0.01 |
| Peritoneal metastasis |  |  |  |
| Yes (vs. No) |  | 1.75 (1.42–2.14) | < 0.01 |

CI, confidence interval; ECOG PS, Eastern Cooperative Oncology Group Performance Status; HER2, human epidermal growth factor receptor 2; HR, hazard ratio
